# Demographic Diversity and Sustainable Fisheries

**DOI:** 10.1371/journal.pone.0034556

**Published:** 2012-05-01

**Authors:** Masami Fujiwara

**Affiliations:** Department of Wildlife and Fisheries Sciences, Texas A&M University, College Station, Texas, United States of America; University of Alberta, Canada

## Abstract

Fish species are diverse. For example, some exhibit early maturation while others delay maturation, some adopt semelparous reproductive strategies while others are iteroparous, and some are long-lived and others short-lived. The diversity is likely to have profound effects on fish population dynamics, which in turn has implications for fisheries management. In this study, a simple density-dependent stage-structured population model was used to investigate the effect of life history traits on sustainable yield, population resilience, and the coefficient of variation (CV) of the adult abundance. The study showed that semelparous fish can produce very high sustainable yields, near or above 50% of the carrying capacity, whereas long-lived iteroparous fish can produce very low sustainable yields, which are often much less than 10% of the carrying capacity. The difference is not because of different levels of sustainable fishing mortality rate, but because of difference in the sensitivity of the equilibrium abundance to fishing mortality. On the other hand, the resilience of fish stocks increases from delayed maturation to early maturation strategies but remains almost unchanged from semelparous to long-lived iteroparous. The CV of the adult abundance increases with increased fishing mortality, not because more individuals are recruited into the adult stage (as previous speculated), but because the mean abundance is more sensitive to fishing mortality than its standard deviation. The magnitudes of these effects vary depending on the life history strategies of the fish species involved. It is evident that any past high yield of long-lived iteroparous fish is a transient yield level, and future commercial fisheries should focus more on fish that are short-lived (including semelparous species) with high compensatory capacity.

## Introduction

Organisms exhibit a wide range of life history strategies [1]. For example, some have early maturation while others delay maturation, some adopt semelparous reproductive strategies while others are iteroparous, and some are long-lived and others short-lived. Such demographic diversity is likely to have profound effects on population dynamics. As fisheries management worldwide faces the challenge of managing fish stocks that encompass broad demographic diversity, there is great interest in investigating the relationship between life history traits and population dynamics [2], [3], [4], [5], [6], [7], and the need to adjust fish stock management based on fish life history strategies, e.g. [8], [9], [10], [11], [12]. In this study, a simple population model was used to assess the effects of demographic diversity on population dynamics under fishing mortality.

One of the most important concepts in fishery management is sustainable yield, see [13], [14], [15]. In a general sense, sustainable yield is a consistent catch over a long (often infinite) period of time, e.g. [16] and often equated to the catch level that results in a stable equilibrium abundance under a deterministic fishery model. When it is at the maximum level, the sustainable yield is called the maximum sustainable yield. When a model-based MSY is available, the desirable yield or fishing mortality can be determined after incorporating precautionary measures that reflect uncertainties, including fluctuations in the environment, errors in parameter estimates, and deficiencies in model formulations [17]. As sustainable yield plays an important role in fishery management, I investigate how sustainable yield and MSY varies with different life history traits of target fish and the fishing mortality rate.

Another objective of this study was to investigate how the sensitivity of transient population dynamics is affected by the life history traits of fish and the fishing mortality rate. Understanding transient dynamics is important in the management of natural resources [18], [19], [20] because a large part of what we actually observe are transient dynamics. In this study, transient population dynamics are measured in two ways. The first involves estimating the coefficient of variation (CV) of adult fish abundance [21] under stochastically fluctuating juvenile survival, which is commonly thought to be the fish population parameter most sensitive to fluctuating environmental conditions. A recent study demonstrated that increased fishing mortality also increases the CV of adult fish abundance [21]. There is great interest in understanding how this measure is affected by various factors, e.g. [22] because unpredictability associated with a large fluctuation in fish abundance will reduce the optimal fishing quota [23]. The second measure of transient dynamics involves calculation of the resilience of the population abundance near a stable equilibrium point. Resilience is a measure of the time that takes for a population to return to asymptotic dynamics after a perturbation, e.g. [24], [25], [26]. Resilience is a measure of intermediate-term transient dynamics, whereas the CV of adult fish abundance is a measure of short-term transient dynamics. In this study, the CV of adult fish abundance, resilience, and MSY were used to characterize short-, intermediate-, and long-term dynamics, respectively, of fishery models incorporating demographic diversity.

In addition to life history traits, a major factor affecting population dynamics is density- dependent regulation, which makes model equations non-linear. Density dependence is necessary for fishery sustainability, and the processes involved have been the subject of much research since Verhulst [27] developed the logistic model. Theoretical understanding of the potential dynamics that can arise from deterministic density-dependent population models is well established. The dynamics converge asymptotically to a stable equilibrium, cycle, aperiodic loop, or chaotic attractor, e.g. [28], [29], [30]. Two types of density- dependent processes are commonly used in fisheries population models. The first is an over-compensatory density-dependent process represented by the Ricker model [31], and the second is compensatory process represented by the Beverton-Holt model [32]. The focus of this study was on density-dependent regulation that results from resource limitation (e.g. competition for available food). This type of regulation is likely to be more common and tends to lead to the Beverton-Holt density-dependent process. Furthermore, the Beverton-Holt density dependence is discrete-time equivalent to the classic logistic model.

## Methods

### Model

The aim of the study was to investigate the effect of demographic diversity on transient and asymptotic population dynamics under various fishing mortality rates, and involved the use of a density-dependent two-stage matrix population model. Although the model is simple, it can incorporate a wide range of life history traits by varying parameter values. In particular, when the life history is semelparous, the model is discrete-time equivalent to a simple logistic (Shaffer) model, which is still widely used in fishery modeling. Therefore, the model presented herein is more general and applicable to real fish populations than the majority of existing fishery population models. Finally, because of the simplicity, asymptotic abundance, sustainable yield, MSY, and resilience can be calculated analytically. This allowed exploration of the model under a wide range of parameter values.

**Figure 1:**
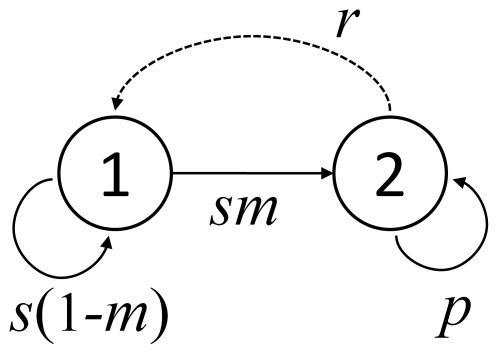
Lifecycle graph of a two-stage model with stage 1 for juveniles and stage 2 for adults. Arrows indicate possible contributions of one stage to the other. *s*: annual survival rate of juveniles, *m*: annual maturation rate, *p*: annual survival rate of adults, and *r*: annual fertility rate.

**Table 1:** Juvenile and adult stage duration after the first reproduction under the parameter values considered in the analysis.

		*m* = 0.1			*m* = 0.5			*m* = 0.9		
		Figure Panel	Juvenile Duration (years)	Adult Duration(years)	Figure Panel	Juvenile Duration (years)	Adult Duration (years)	Figure Panel	Juvenile Duration (years)	Adult Duration (years)
*s* = 0.1	*p* = 0.0	4a	1.10	1	4b	1.05	1	4c	1.01	1
	*p* = 0.5	4d	1.10	2	4e	1.05	2	4f	1.01	2
	*p* = 0.9	4g	1.10	10	4h	1.05	10	4i	1.01	10
*s* = 0.5	*p* = 0.0	3a	1.82	1	3b	1.33	1	3c	1.05	1
	*p* = 0.5	3d	1.82	2	3e	1.33	2	3f	1.05	2
	*p* = 0.9	3g	1.82	10	3h	1.33	10	3i	1.05	10
*s* = 0.9	*p* = 0.0	2a	5.26	1	2b	1.82	1	2c	1.10	1
	*p* = 0.5	2d	5.26	2	2e	1.82	2	2f	1.10	2
	*p* = 0.9	2g	5.26	10	2h	1.82	10	2i	1.10	10

The panel indices correspond to those in Figures 2–4. Life histories on Figures 5, 8, and 11 are the same as Figure 2. Life histories on Figures 6, 9, and 12 are the same as Figure 3. Life histories on Figures 7, 10, and 13 are the same as Figure 4.

The model consists of two stages (Fig. 1). The first (the juvenile stage) is for reproductively immature individuals, and the second (the adult stage) is for mature individuals. The model includes four population parameters: juvenile survival rate (*s*), adult survival rate (*p*), maturation rate (*m*), and fertility (*r*). It was assumed that the time unit was one year, and therefore the rates were annual rates. Fertility (*r*) is the product of the survival of adults until the reproductive season and the annual per capita fecundity (the number of eggs). This type of matrix population model is called a post-breeding model [33], in which the population abundance is determined immediately after spawning event. Because the survival rate of eggs and juveniles are often substantially different for fish, it is assumed that the multiplicative difference between the egg and juvenile survival is also implicitly included in *r*. When the four parameters in the model are density independent, the densities of juveniles (*n*_1_) and adults (*n*_2_) are “projected” from year *t* to the next year, as follows:
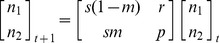
(1)where the subscripts of the vectors denote year. The matrix is in general termed a population matrix. The dominant eigenvalue of the matrix gives the annual asymptotic population growth rate, and an associated right eigenvector gives the relative asymptotic densities between the two stages, see [31].

**Figure 2:**
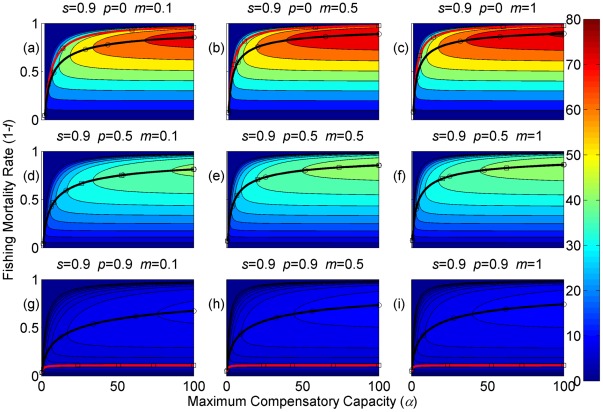
Sustainable yield (shown in contours) as a function of the annual fishing mortality rate and the maximum compensatory capacity when *s* = 0.9. The black curves with circle markers indicate the annual fishing mortality rate at the maximum sustainable yield as a function of the maximum compensatory capacity. The red curves with square markers indicate the annual fishing mortality rate when the equilibrium adult abundance is 50% of the carrying capacity (i.e. asymptotic adult abundance when there is no fishing mortality) as a function of the maximum compensatory capacity. Each panel represents a life history strategy defined by the parameters shown above.

**Figure 3 pone-0034556-g003:**
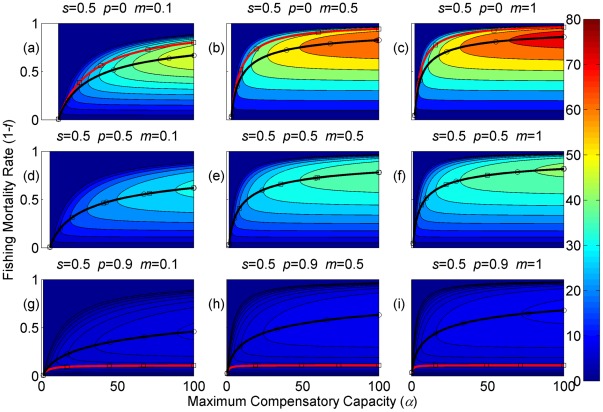
Sustainable yield (shown in contours) as a function of the annual fishing mortality rate and the maximum compensatory capacity when *s* = 0.5. The black curves with circle markers indicate the annual fishing mortality rate at the maximum sustainable yield as a function of the maximum compensatory capacity. The red curves with square markers indicate the annual fishing mortality rate when the equilibrium adult abundance is 50% of the carrying capacity (i.e. asymptotic adult abundance when there is no fishing mortality) as a function of the maximum compensatory capacity. Each panel represents a life history strategy defined by the parameters shown above.

**Figure 4:**
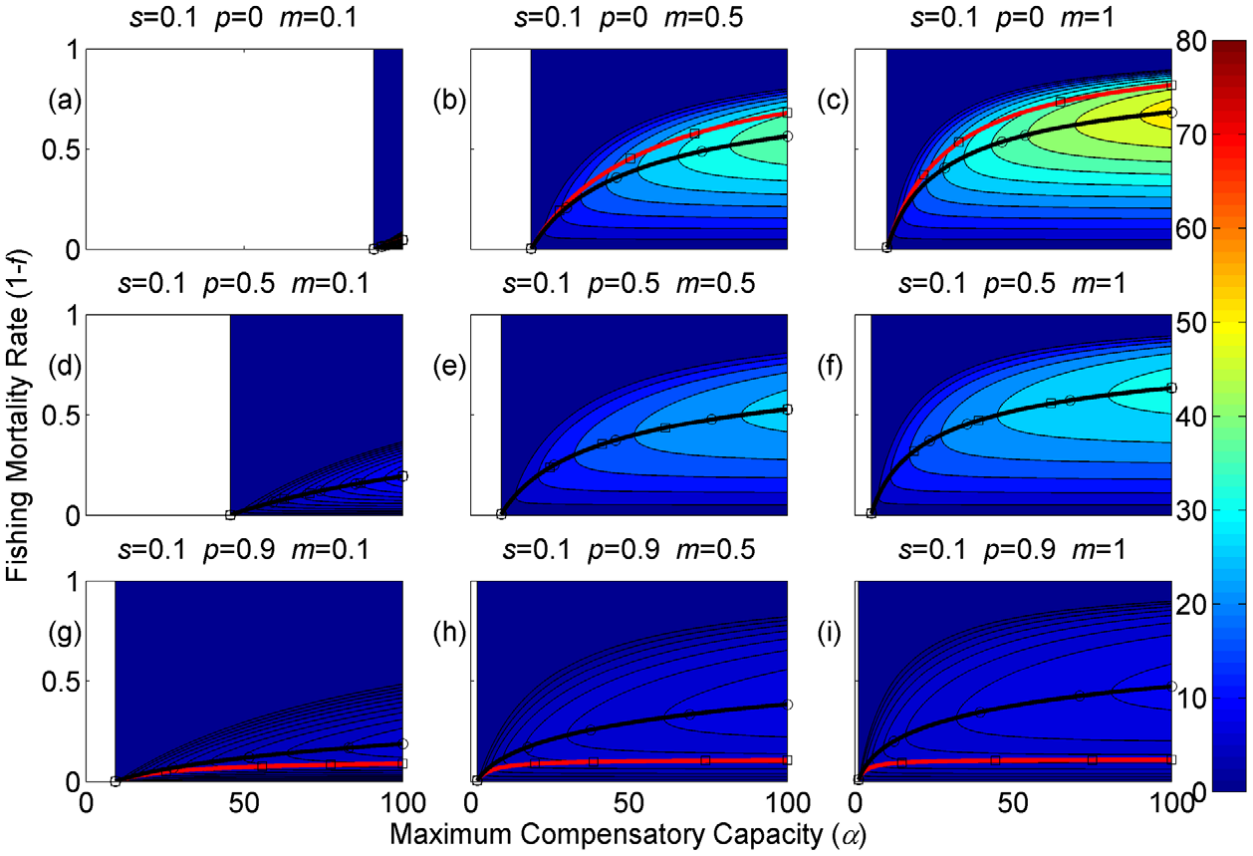
Sustainable yield (shown in contours) as a function of the annual fishing mortality rate and the maximum compensatory capacity when *s* = 0.1. The black curves with circle markers indicate the annual fishing mortality rate at the maximum sustainable yield as a function of the maximum compensatory capacity. The red curves with square markers indicate the annual fishing mortality rate when the equilibrium adult abundance is 50% of the carrying capacity (i.e. asymptotic adult abundance when there is no fishing mortality) as a function of the maximum compensatory capacity. Each panel represents a life history strategy defined by the parameters shown above.

**Figure 5:**
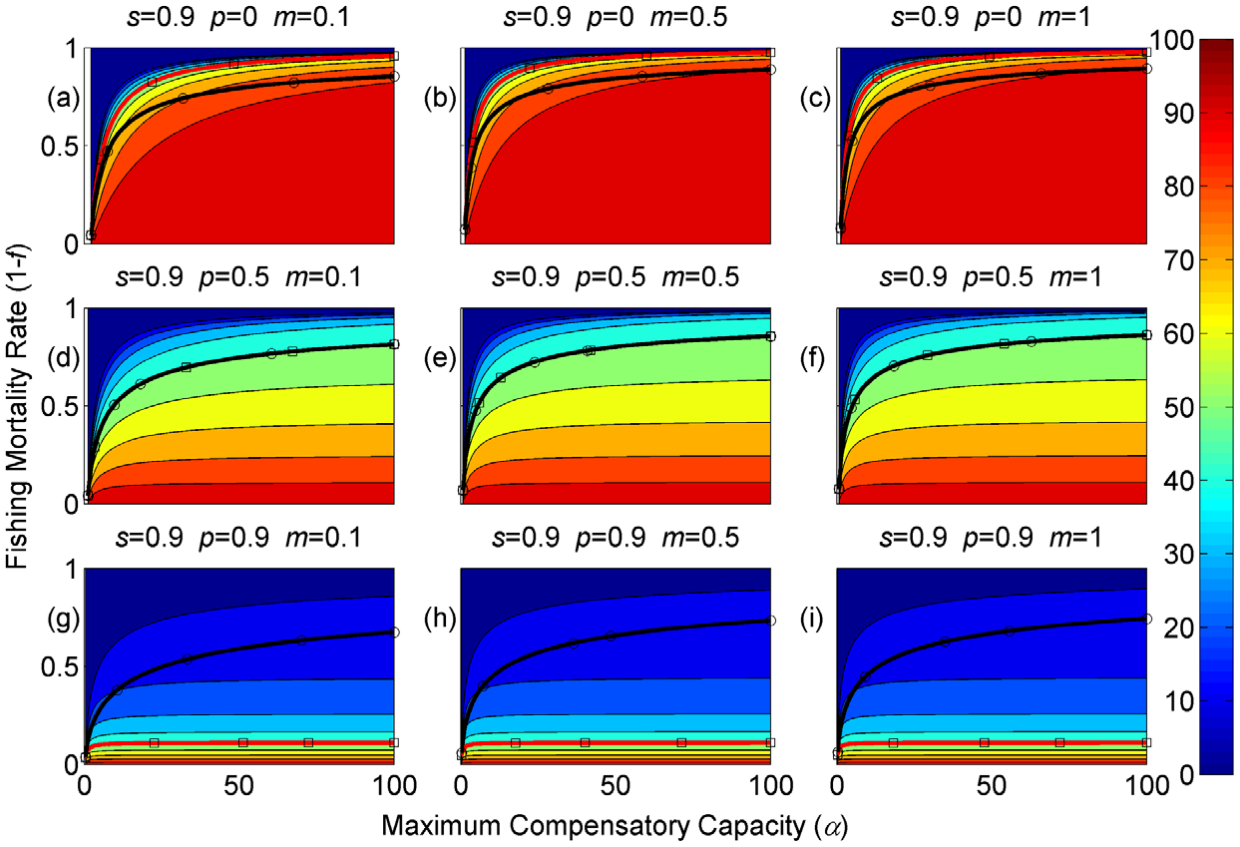
Equilibrium adult abundance (shown in contours) as a function of the annual fishing mortality rate and the maximum compensatory capacity when *s* = 0.9. The black and red curves are the same as shown in Figure 2. Each panel represents a life history strategy defined by the parameters shown above.

**Figure 6:**
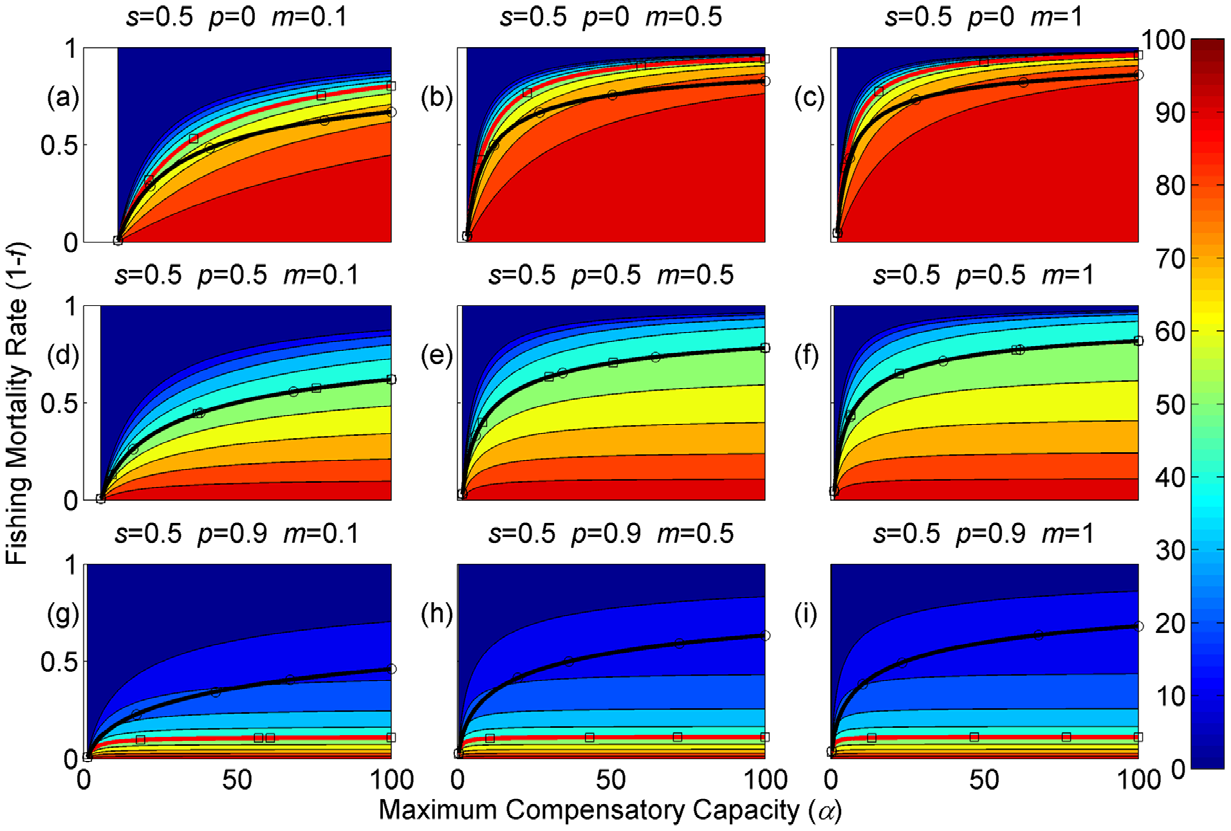
Equilibrium adult abundance (shown in contours) as a function of the annual fishing mortality rate and the maximum compensatory capacity when *s* = 0.5. The black and red curves are the same as shown in Figure 3. Each panel represents a life history strategy defined by the parameters shown above.

**Figure 7:**
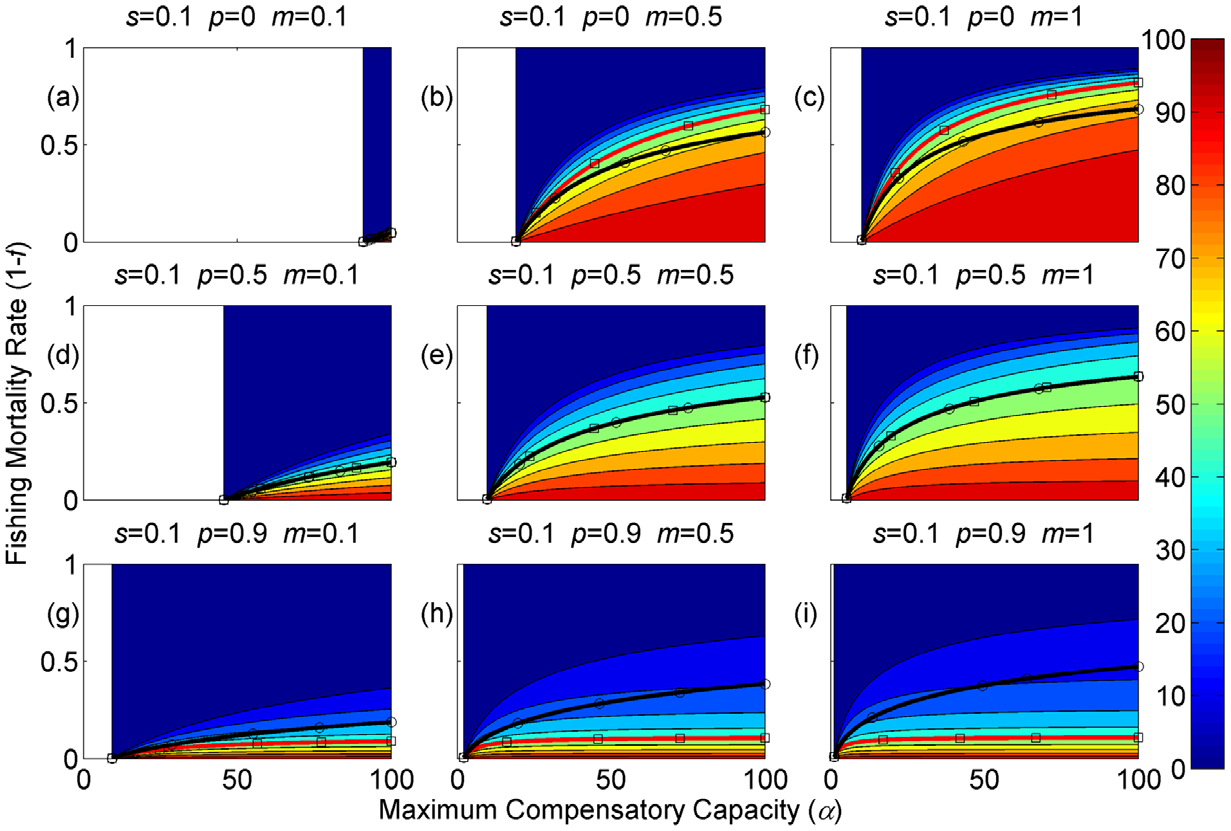
Equilibrium adult abundance (shown in contours) as a function of the annual fishing mortality rate and the maximum compensatory capacity when *s* = 0.1. The black and red curves are the same as shown in Figure 4. Each panel represents a life history strategy defined by the parameters shown above.

By varying parameter values, various life history strategies can be incorporated into the model. For example, by increasing *m* from a low to a high value, the life history strategy is modified from maturing early (precocious) to maturing late (delayed maturation). Together *m* and *s* determine the mean age of maturation. By increasing *p* from 0 toward 1 the life history strategy changes from semelparous to iteroparous. It is noted that even if *p* = 0, a positive value of *r* ensures that some individuals will reproduce once before their death. The generation time of the organisms is determined by *m*, *s* and *p*. Under this model, the average time individuals spend in the juvenile stage before maturing is given by 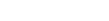, and the average time individuals spend in the adult stage is given by 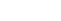. These average stage durations can be used to approximate actual species of fisheries interest using modeled life history strategies.

Fishing mortality affects adult survival. It is incorporated into the model by multiplying the rate of surviving from fishing mortality (*f *) with parameters in the projection matrix. Thus, 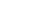
is the annual fishing mortality rate. With fishing mortality included, the equation becomes:
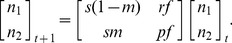
(2)The fertility term is also multiplied by *f* because *r* includes the adult survival rate as described previously. The sequence of event is that (1) fishing mortality and natural adult mortality and then (2) spawning. The use of a post-breeding matrix population model avoids spurious results in which a population is persistent with the adult fishing mortality rate of 1 because newly recruited adults always have at least one chance of reproduction. An alternative way of modeling is to have a pre-breeding model, and the <2,1> entry of the population matrix is multiplied by *f* instead of the <1,2> entry.

**Figure 8:**
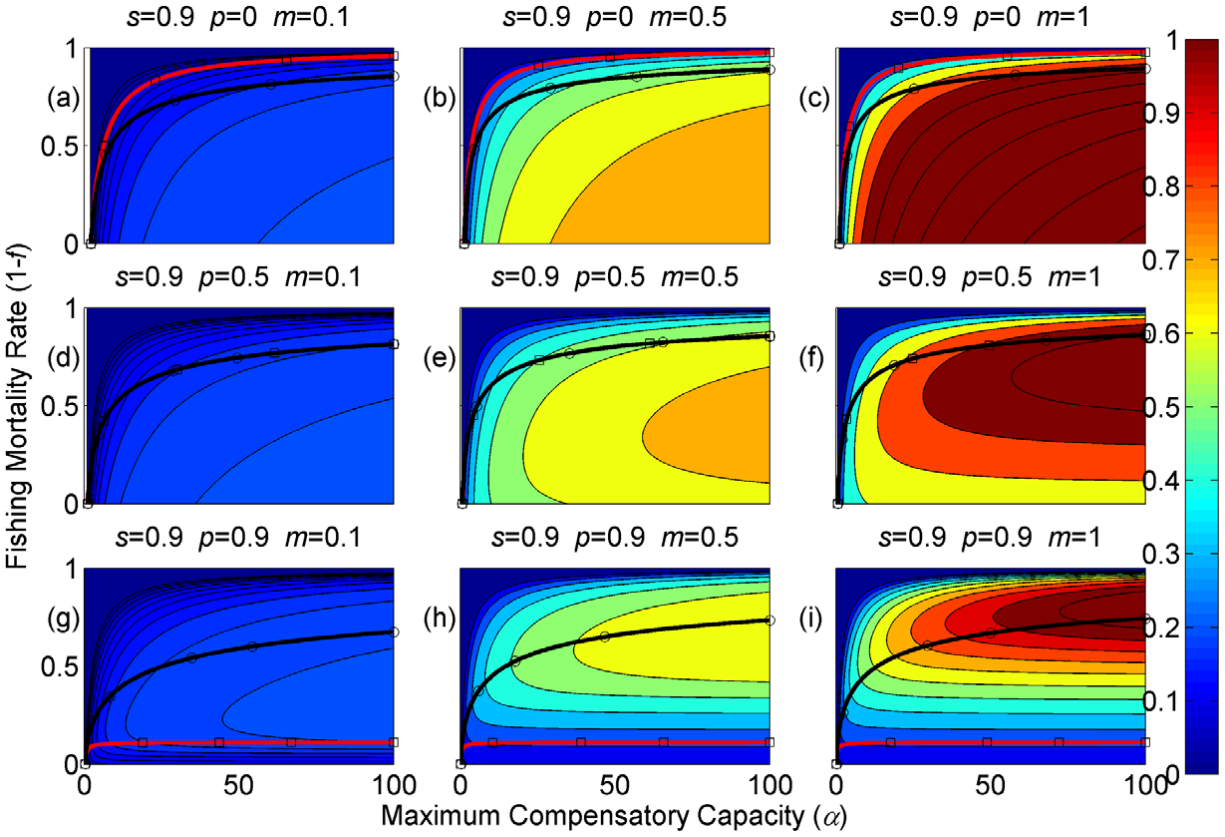
Resilience of the equilibrium densities (shown in contours) as a function of the annual fishing mortality rate and the maximum compensatory capacity when *s* = 0.9. The black and red curves are the same as shown in Figure 2. Each panel represents a life history strategy defined by the parameters shown above.

**Figure 9:**
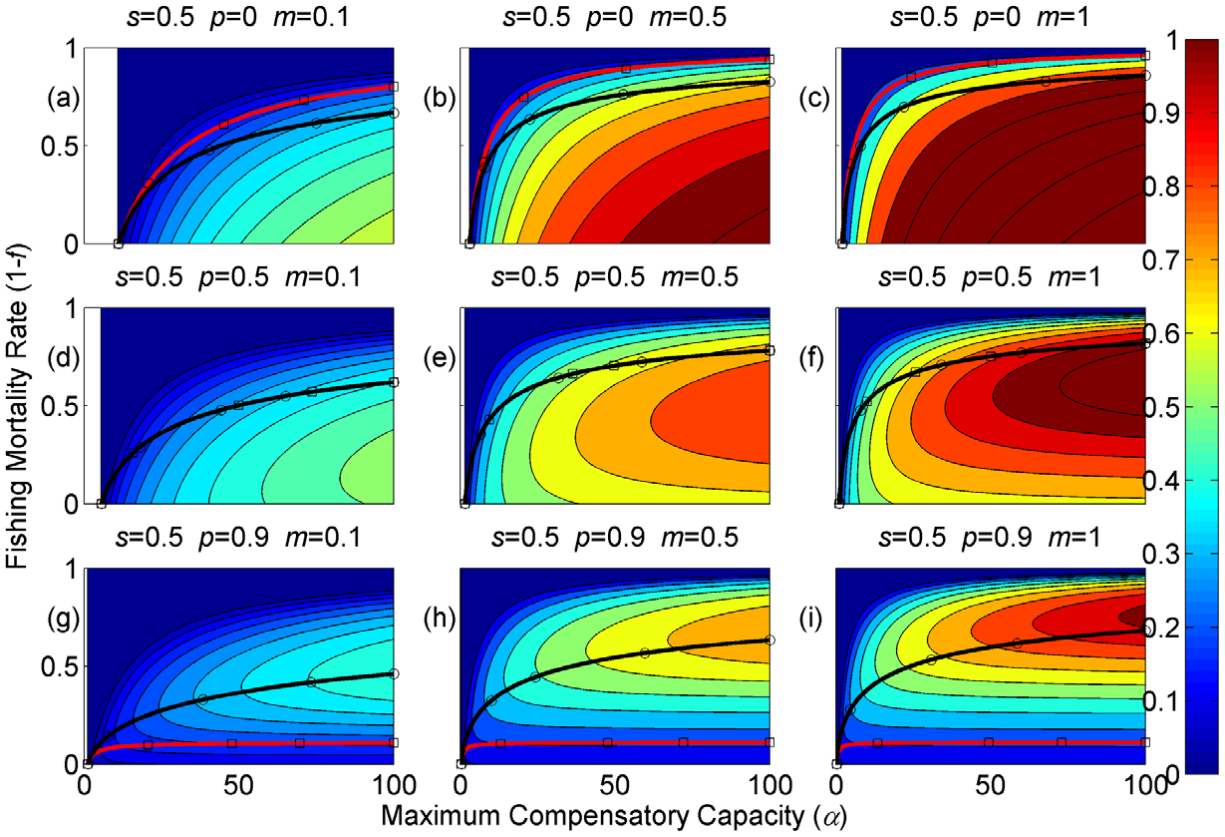
Resilience of the equilibrium densities (shown in contours) as a function of the annual fishing mortality rate and the maximum compensatory capacity when *s* = 0.5. The black and red curves are the same as shown in Figure 3. Each panel represents a life history strategy defined by the parameters shown above.

**Figure 10:**
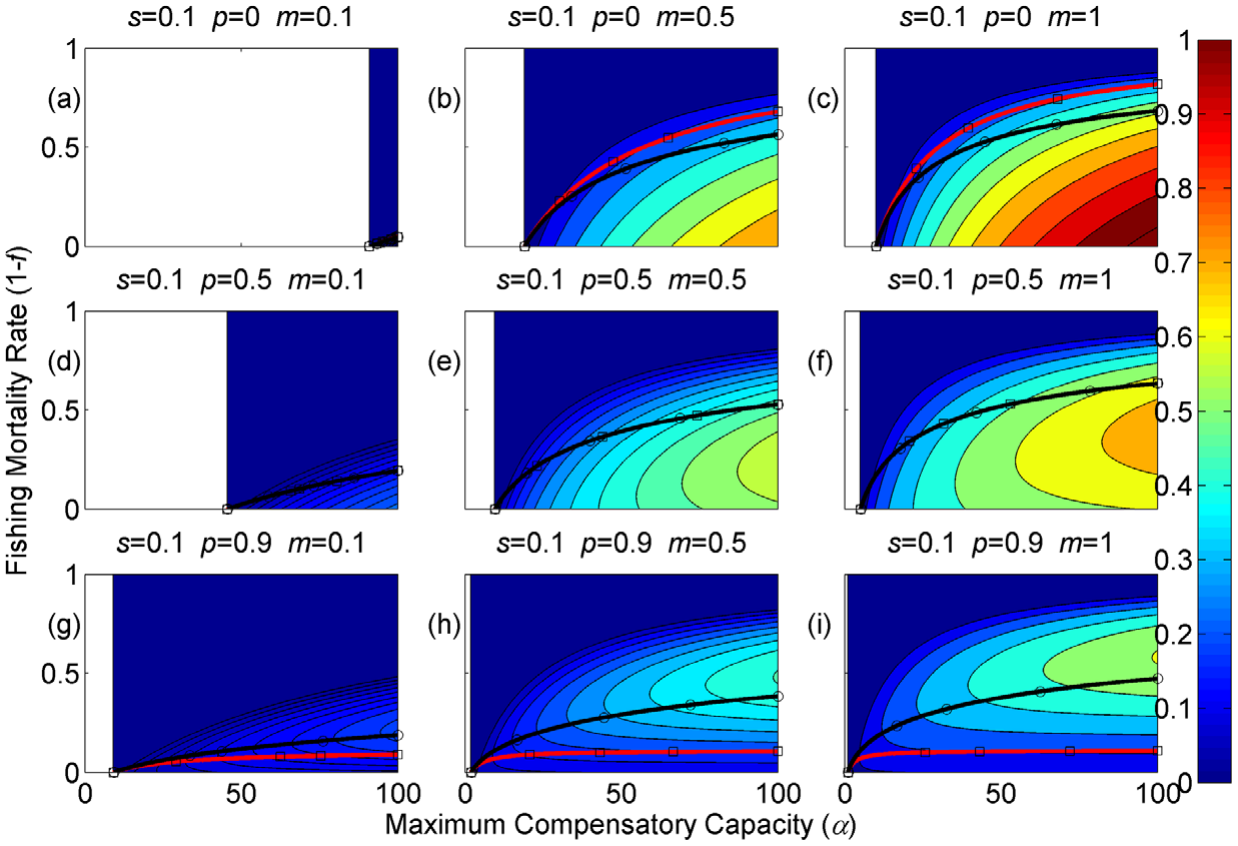
Resilience of the equilibrium densities (shown in contours) as a function of the annual fishing mortality rate and the maximum compensatory capacity when *s* = 0.1. The black and red curves are the same as shown in Figure 4. Each panel represents a life history strategy defined by the parameters shown above.

The Beverton-Holt density-dependent process is also incorporated into the model. Here, it was assumed that the effect of the density-dependent process is on fecundity and/or the survival during the first year of life. Therefore, it was incorporated into the fertility term, which includes both fecundity and the difference in survival rate between egg and juvenile stage, as:
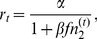
(3)which replaces *r* in equation (2). Parameter *α* is the maximum per capita fertility rate at a low adult abundance, and it is referred to here as the maximum compensatory capacity (*α*). For a given value of *α*, parameter *β* determines the equilibrium adult abundance. It can be shown that under this model if there is an equilibrium point at which both juvenile and adult densities are positive, the equilibrium point is stable.

In equation (1), there are four population parameters. However, under the stable equilibrium point the dominant eigenvalue of the population matrix is 1. Therefore, the number of free population parameters is three under the equilibrium point without fishing mortality, and consequently the fertility rate at the equilibrium point was chosen to be varied as a function of *m*, *s* and *p*. Thus, by setting the eigenvalue to 1, the fertility term at the equilibrium under no fishing mortality (r*) can be solved as follows.
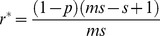
(4)


There are two density-dependent parameters (*α* and *β*) in equation (3). However, for given values of *α*, r* and an equilibrium adult abundance 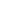, parameter *β* is expressed as:
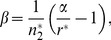
(5)when fishing mortality rate is 0 (*f* = 1). In the subsequent analyses, the equilibrium adult abundance without any fishing mortality was set at 100 under all life history traits. Hereafter, this level is referred to as the carrying capacity. Setting the carrying capacity at 100 allows any reduction in the equilibrium abundance because of fishing mortality to be interpreted as a percent decline from the carrying capacity. Similarly, sustainable yield can also be related to the percent of the carrying capacity.

The use of a simple model means various simplifying assumptions were made. For example, instead of modeling both biomass and fish abundance (common practices in fishery modeling), the focus was placed solely on the latter. Similarly, instead of categorizing fish into different sizes as well as maturation status, the model only incorporates two stages; juveniles and adults. Consequently, although the model can incorporate a wide range of fish life history strategies, it does not incorporate all strategies. Instead, the model was designed to present a broad picture of the population dynamics of fish over a wide range of life history strategies, thus complementing existing efforts to build and analyze more complex fishery models, which are often stock specific.

The model in this study is closely related to stock recruitment/replacement-line models, which are frequently used in fisheries management, e.g. [14], [15]. In the current model, the stock recruitment relationship, e.g. [34] is given by the relationship between 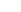 (stock) and 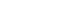
(new recruitment). The slope (often the inverse of the slope) of the replacement line, which is stock abundance per new recruit, is given by the ratio between 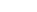 and 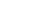. However, the model includes population parameters more explicitly, enabling the investigation of life history variations.

The model is also closely related to the surplus production model, which is often expressed in terms of ordinary differential equations. For example, the most basic surplus production model uses a logistic growth model with added instantaneous fishing mortality, see [14], [35]. The current model is equivalent to the surplus production model except it includes life history strategy of fish and it is modeled with life history strategy events including reproduction, compensatory density dependence, survival, maturation and fishing mortality occurring in a discrete sequence rather than simultaneously. The Beverton-Holt density dependence is also a discrete-time equivalent to the density dependence under the logistic equation.

### Analyses

The ways in which equilibrium fishery yield, resilience, and the CV of adult abundance responded to changes in the parameters in the model (i.e. life history traits) were investigated. In particular, *s*, *m* and *p* were varied among low, intermediate and high values (*s*: 0.1, 0.5, 0.9; *m*: 0.1, 0.5, 1.0; *p*: 0, 0.5, 0.9). Average stage durations were calculated for each combination of parameters in the absence of fishing mortality. The equilibrium fishery yield, resilience, and CV of adult abundance were then calculated over ranges of *α* and fishing mortality (1−*f*), as described below.

Fishery yield at equilibrium (Y^*^) is given by
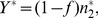
(6)where 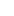
is the equilibrium adult abundance under a fishing morality rate of 1−*f*. The expression for the equilibrium adult abundance is obtained from equations (2) and (3) as
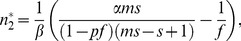
(7)by setting the dominant eigenvalue of the population matrix (2) to be 1 and solving the equation for the adult abundance.

The fishing mortality rate at MSY satisfies the equation that is derived by taking the derivative of Y^*^ with respect to *f*, and setting the derivative to 0. In equation (6), 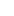 is also a function of *f*, and consequently the expression for the fishing mortality rate at MSY is not simple. However, the procedure for analytically taking the derivative is straightforward. The fishing mortality rate at MSY can be substituted into equations (6) and (7) to obtain the MSY.

The resilience (R) of a density-dependent matrix population model around the equilibrium point measures how quickly a population returns to an equilibrium point after a perturbation, and it is given by the negative slope of a log transformed difference between population abundance and the equilibrium point approaching 1. It is given by:
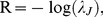
(8)where 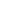
is the absolute value of the dominant eigenvalue of a linearized population projection matrix around the equilibrium point [33]. The linearized population projection matrix in this study was:



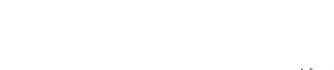
where * indicates that the population is at the equilibrium point and all of the derivatives are also evaluated at the equilibrium point. The linearized matrix is similar to the original population matrix because only one of the four parameters experience density dependence in the model. As it is a 2×2 matrix, obtaining the dominant eigenvalue of this matrix was also straightforward.

The CV of adult abundance [21] was estimated by incorporating stochastic perturbations into *s*, and simulating the population over 1000 time steps starting from the equilibrium abundance of a deterministic model. In this simulation, *s* was allowed to fluctuate according to the Beta distribution with means of 0.1, 0.5, and 0.9, and a constant variance of 0.01, without any serial autocorrelation. After each 1000 time step simulation, the CV of adult abundance was estimated.

## Results

The average stage durations of various life history traits considered in this study are shown in Table 1 for reference. Hereafter, species with *p* = 0.0, 0.5, and 0.9 are referred to as semelparous, short-lived iteroparous, and long-lived iteroparous, respectively. Similarly, species with a juvenile stage longer than 1.5 years are referred to as having delayed maturation, while others are considered to be precocious.

Asymptotic fishery yields as a function of fishing mortality and the maximum compensatory capacity are shown in the contours in Figures 2, 3, 4 (for *s* = 0.9, 0.5, and 0.1, respectively). For example, Figure 2a shows asymptotic fishery yield for one of the semelparous precocious life history strategies. For a given value of maximum compensatory capacity, the sustainable yield initially increases as fishing mortality is increased from 0. This is because fishers catch more with increased effort. However, it peaks at some intermediate value of fishing mortality and declines thereafter because the equilibrium abundance of adults declines rapidly with increased mortality when the mortality rate is high. The grey area in the left side of some panels indicates that the population is not sustainable even without fishing mortality because of a low maximum compensatory capacity. As we go down the panels in each figure, life history is semelparous (*p* = 0) short-lived interoparous (*p* = 0.5), and long-lived iteroparous (*p* = 0.9). As we move from the left to right panels, maturation rate increases.

The black curves with circles show the level of fishing mortality at the maximum sustainable yield for a given value of maximum compensatory capacity. As maximum compensatory capacity is increased, the population can be fished at a higher rate because the losses from the fishery are better compensated for, and the MSY also increases.

The red curves with square markers show the annual fishing mortality rate that reduces the adult abundance to 50% of the carrying capacity (i.e. the equilibrium abundance under no fishing mortality). This mortality level is referred to as the 0.5 *K* mortality. It should be noted, in the model, the equilibrium abundance is measured prior to fishing mortality but after recruitment. Under a traditional surplus production model with a logistic equation and instantaneous yield, which may be constant or proportional to fish abundance, the MSY is achieved when the population abundance is reduced to 50% of the carrying capacity. Figures 2, 3, 4 shows that the MSY fishing mortality is higher than the 0.5 *K* mortality when the survival rate of adults is high (*p* = 0.9). Conversely, the MSY fishing mortality is lower than the 0.5 *K* mortality when the survival rate of adults is low (*p* = 0). They are equal when the survival rate of adults is at the intermediate value (*p* = 0.5) under all juvenile survival and maturation rates.

It is evident that the sustainable yield at a given value of fishing mortality and MSY declines from the semelparous (*p* = 0) to the iteroparous (*p* = 0.5) strategies, and also from short-lived iteroparous (*p* = 0.5) to long-lived iteroparous (*p* = 0.9) strategies. Therefore, the sustainable yield is the property associated with the adult parameter. This is despite the fact that the fishing mortality rate at MSY only slightly decreases from the semelparous to the long-lived iteroparous. For long-lived iteroparous species the maximum sustainable yield is always less than 10% of the carrying capacity, whereas for semelparous species the MSY can be greater than 50% of the carrying capacity under some parameter values. This results from differences in equilibrium abundance under fishing mortality among the various life history strategies.

The dark blue region in the upper left corner in each panel of Figures 2, 3, 4 indicates the region of unsustainable fisheries. As *s* is reduced (i.e. from Figure 2 to 3 and Figure 3 to 4), the region of unsustainable fisheries increases, and this is more pronounced when *m* is low. This occurs because, at a reduced *s*, few individuals are recruited into the adult stage. Consequently, only a slight increase in the adult mortality as a consequence of fishing will cause fishing to become unsustainable.

Equilibrium adult abundance as a function of fishing mortality and maximum compensatory capacity are shown in the contours in Figures 5, 6, 7 (for *s* = 0.9, 0.5, and 0.1, respectively). Figure 5a shows the equilibrium adult abundance for one of the semelparous precocious life history strategies. For a given value of maximum compensatory capacity, as fishing mortality rate is increased, equilibrium adult abundance declines. When maximum compensatory capacity is low, the decline starts early and quickly reaches very low abundance whereas, when maximum compensatory capacity is high, the population can maintain high abundance with higher fishing mortality rate. The black curve with circles and the red curves with squares are the same as before: fishing mortality rate at MSY and 0.5 *K* abundance, respectively. As adult survival rate (i.e. as we go down the panels) is increased, the equilibrium adult abundance declines substantially. On the other hand, maturation rate has almost no effect on the equilibrium adult abundance. These trends remain the same with lower juvenile survival rate (Fig. 6 and 7). However, as juvenile survival rate declines, the equilibrium abundance declines faster with increasing fishing mortality rate.

The resilience of a population around the equilibrium point is shown in Figures 8, 9, 10 (for *s* = 0.9, 0.5, and 0.1, respectively). With low maximum compensatory capacity, the population resilience always declines with increasing fishing mortality. This means that if there is a perturbation to the population, such as a natural or anthropogenic disaster, it will take longer to return to the equilibrium point as fishing mortality increases. Conversely, when maximum compensatory capacity is high, the resilience initially increases with increased fishing mortality, peaks, and then declines. However, irrespective of whether maximum compensatory capacity is high or low, when the fishing mortality exceeds the level for MSY, in most cases, the resilience declines with increasing fishing mortality.

Resilience is also affected by life history traits (Fig. 8, 9, 10). It increases with increasing maturation rate *m* and juvenile survival rate *s.* Although resilience decreases with increasing adult survival rate *p*, the change is by small amount. Therefore, the resilience is the property associated with parameters of juvenile stage. Finally, increased maximum compensatory capacity also increases resilience because of high compensatory capability.

**Figure 11:**
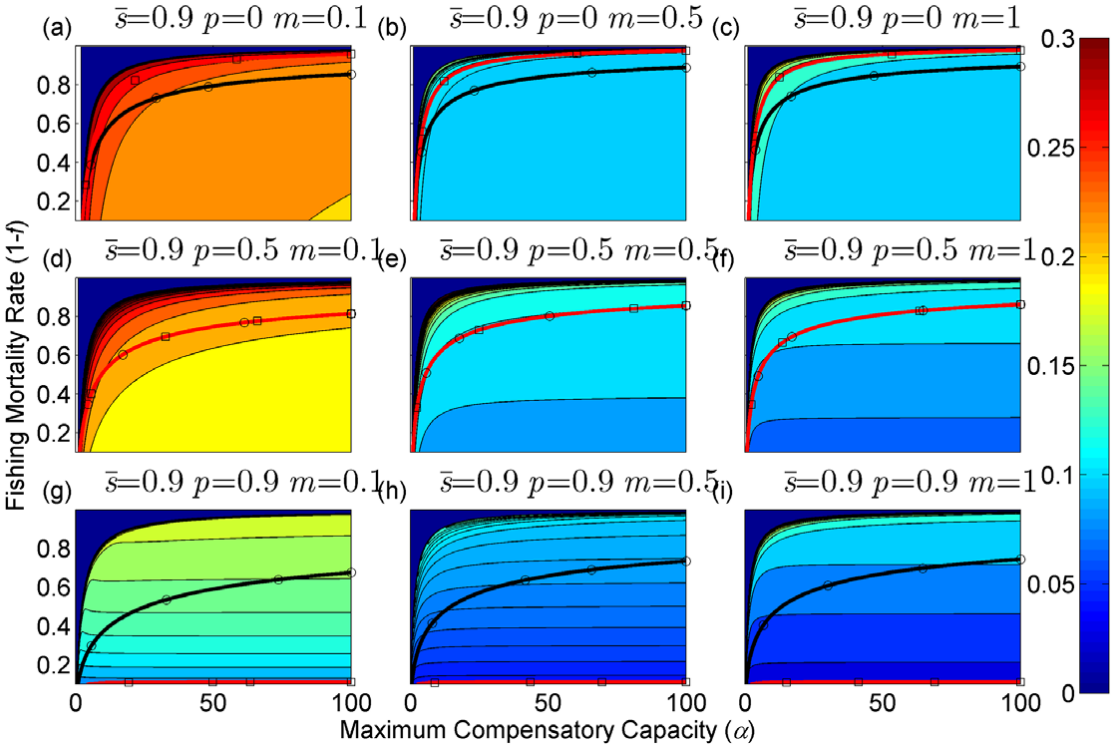
Coefficient of variation (CV) of adult abundance (shown in contours) as a function of the annual fishing mortality rate and the maximum compensatory capacity when juvenile survival fluctuates stochastically when 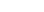. The black and red curves are the same as shown in Figure 2. Each panel represents a life history strategy defined by the parameters shown above.

**Figure 12:**
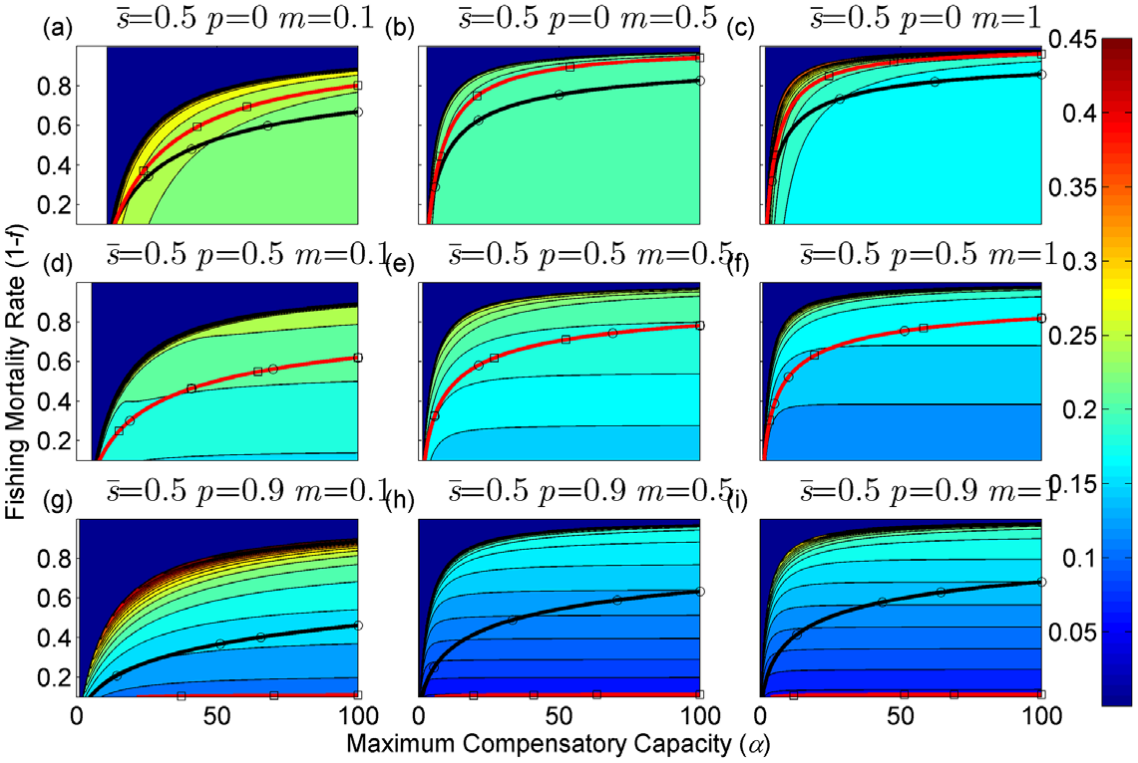
Coefficient of variation (CV) of adult abundance (shown in contours) as a function of the annual fishing mortality rate and the maximum compensatory capacity when juvenile survival fluctuates stochastically when 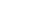. The black and red curves are the same as shown in Figure 3. Each panel represents a life history strategy defined by the parameters shown above.

**Figure 13:**
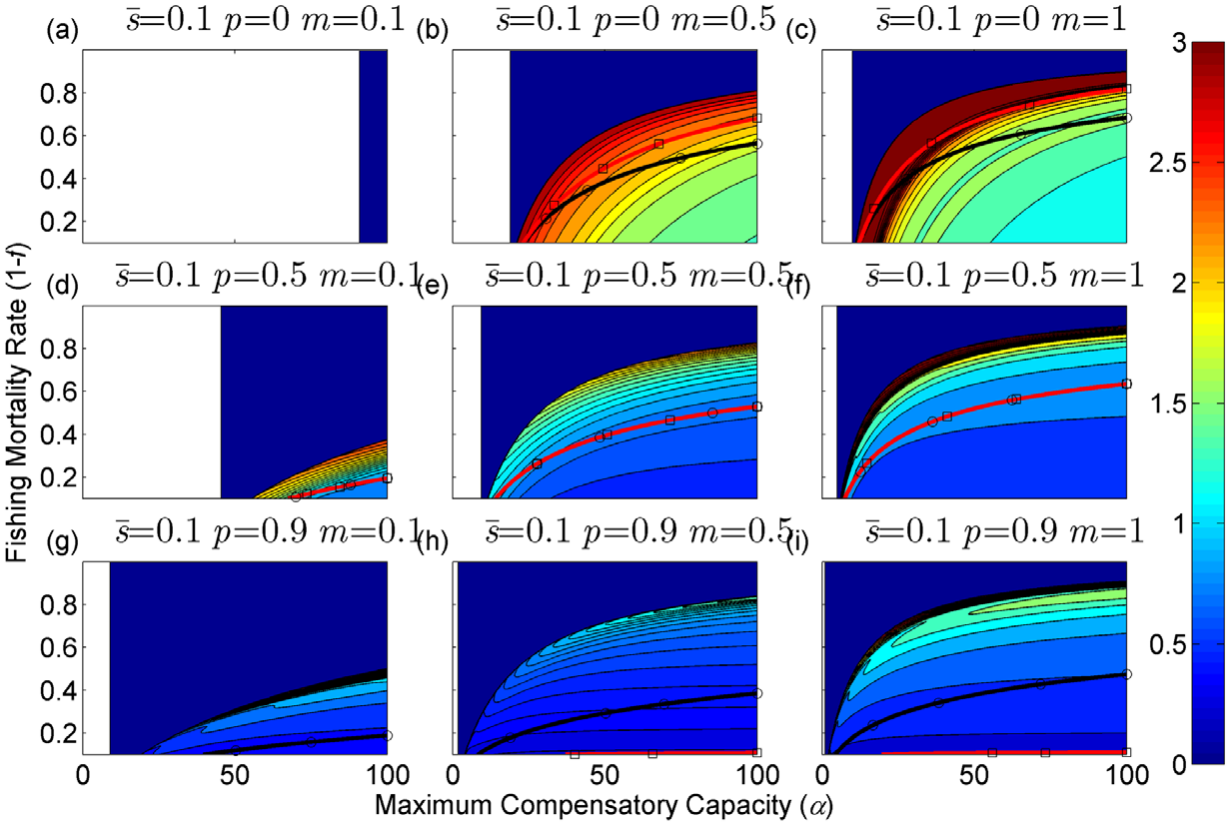
Coefficient of variation (CV) of adult abundance (shown in contours) as a function of the annual fishing mortality rate and the maximum compensatory capacity when juvenile survival fluctuates stochastically when 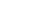. The black and red curves are the same as shown in Figure 4. Each panel represents a life history strategy defined by the parameters shown above.

The CV of the adult abundance increased with increased fishing mortality in all cases (Fig. 11, 12, 13). This implies that the population fluctuates more with higher fishing mortality rate. This occurs regardless of whether the fish have iteroparous, semelparous, precocious, or delayed maturation life history strategies.

## Discussion

A simple stage-structured model was used to investigate how fishing mortality affects the population dynamics of fish with different life history strategies. Although simple, the model can encompass a wide variety of life history strategies. For example, Winemiller and Rose [5] investigated 10 life history traits of 216 North American fish species. The study revealed that the species can be categorized by three attributes: juvenile survivorship, generation time, and fecundity. The model in the present study also included three life history parameters that were varied to represent demographic diversity. Juvenile survival was explicitly included in the model, and generation time and fertility (instead of fecundity) were also functions of the three parameters in the model. Therefore, similar life history variation was investigated in the current study, but was parameterized in different ways. Fecundity (number of eggs produced) was not explicitly included in the model because the number of eggs that a fish produces is often a local/regional adaptation to the environment [36]. Of more importance to the overall population dynamics is the product of the survival rate of adults over the time-scale of a population model (1 year in the model in this study) and their fecundity (or fecundity and the survival of offspring over the time-scale of a population model in pre-breeding model).

The results suggest that the sustainable yield is reduced from long-lived iteroparous species (panels g, h, i in Fig. 2, 3, 4) to short-lived iteroparous species (panels d, e, f in Fig. 2, 3, 4) and also from short-lived iteroparous to semelparous (panels g, h, i in Fig. 2, 3, 4) under the same fishing mortality rate. This results from reduced adult equilibrium abundance, and suggests that the sensitivity of the equilibrium abundance increases as the duration of an adult stage increases. This means that, although we can rebuild over-exploited longer-lived fish by stopping its exploitation, after resuming exploitation, population abundance will decline again even with a low level of fishing mortality rate.

The MSY also exhibits large differences among different life history strategies. For example, there is a large difference in the MSY between long-lived iteroparous and semelparous fish (Fig. 2, 3, 4). For long-lived iteroparous species, the MSY was always less than 8% (often much less than that) of the carrying capacity. This result suggests that the large yields that fishers may have obtained in the past with some long-lived fish are transient yield and cannot be sustained at the same level. The only way to achieve those yields (although still transiently) is to rebuilt the stocks to historical levels. It is possible that the low MSY for long-lived iteroparous species will not be economically viable for many fishery stocks. However, the MSY of semelparous fish can be 10 fold greater than that of long-lived iteroparous species under the same fishing mortality.

In contrast to MSY and sustainable yield, fishing mortality rate at MSY was similar among different life history strategies (Fig. 2, 3, 4). Semelparous fish have slightly higher fishing mortality at MSY than short-lived iteroparous species, and short-lived iteroparous fish have slightly higher fishing mortality at MSY than long-lived iteroparous species. However, the differences are small, and the factor that is differentially affected by fishing mortality among different life history strategies is the equilibrium abundance of adults. If we only want to know the model-based prediction of fishing mortality rate at MSY, it is not necessary to incorporate life history of organisms into the analysis. The information on the compensatory capacity of a fish population is sufficient. However, this does not mean that we should fish at the level of model-based MSY because, when population abundance is suppressed to a very low level, other factors such as environmental fluctuation and depensatory processes may affect the population.

In general, fishing mortality rate at MSY is the additional mortality rate that achieves the maximum production of the population. Under the logistic equation (Schaffer) model, it happens to be the level that suppresses the equilibrium abundance to a half of the carrying capacity (the 0.5 *K* level). However, under the two-stage model with the Beverton-Holt density dependence affecting a fertility term, mortality rates at 0.5 *K* and MSY levels are different. The exception was when the adult natural mortality rate was 0.5 (Fig. 2, 3, 4), but it should be noted that this exception is specific to the model used in the study. Therefore, in general, it cannot be assumed that MSY is achieved when the population abundance is at the 0.5 *K* level.

The maximum compensatory capacity (*α*) of a population affects the sustainable yield of fish; the greater *α* is, the greater the maximum sustainable yield is (Fig. 2, 3, 4). This makes intuitive sense, because, if *α* is high, a reduction in abundance because of fishing mortality can be better compensated for, and in turn allows higher fishing mortality rate. The mortality rate at MSY is also affected more by *α* than other parameters. Furthermore, a model for competition between stage-structured populations suggests the importance of this parameter [37]. The current result re-emphasizes the importance of accurately estimating density-dependent parameters, e.g. [38]. Unfortunately, *α* is probably the parameter in the model that is most difficult to estimate from field observations.

The effects of juvenile survival and maturation rate appear to have synergetic effects. For example, when the juvenile survival or maturation rate is high, reducing the other population parameter has only a small effect on the sustainable yield (Fig. 2, 3, 4). When the maturation rate is low, individuals tend to remain immature for longer. However, the low maturation rate is compensated for by increased reproduction. Consequently, individuals will accumulate in the juvenile stage as long as juvenile survival is high, and greater abundance in the juvenile stage will result in enough number of individuals maturing each year to maintain a population level. Similarly, when juvenile survival is low, a smaller proportion of individuals will survive to maturity, but reduced survival is compensated for by increased reproduction. As long as the maturation rate is sufficiently high, the compensation is sufficient for enough number of individuals to mature each year to maintain equilibrium abundance. However, when juvenile survival or the maturation rate is low, reducing the other parameter has a pronounced effect on the sustainable yield. When both rates are low the population tends to be nonviable even though adults had high fertility rate, which is evidenced by the large grey areas in the figures.

The resilience of a population is an important population dynamics characteristic in fishery management (Fig. 8, 9, 10). The model suggests that fish with relatively low maximum compensatory capacity or those that are caught at levels above the MSY will have reduced resilience as fishing mortality is increased. This means that if there are additional mortalities caused by natural or anthropogenic events, these effects will last longer when the species are subject to high fishing mortality. This can have severe effects on economic sustainability of fisheries. Resilience is not a factor that is commonly incorporated into fishery management. I suggest it should be considered in future management decisions.

Resilience is also affected by life history traits, and fish stocks that exhibit the most resilience are those with an early maturing semelparous life history strategy and a high maximum compensatory capacity. Fish with such life history strategies will return to the equilibrium abundance more rapidly following a perturbation than stocks with a different life history strategy. Long-lived iteroparous species have the lowest resilience, but a change from short-lived iteroparous to long-lived iteroparous life history does not appear to change the resilience as much as the reduction that results from the change from delayed maturation to precocious reproductive strategies. This suggests that resilience is a quality of strategies associated with the juvenile stage. Thus, long-lived fish can have high resilience if they mature early and produce a large number of offspring. In order to determine the speed of recovery of over-exploited fish populations, we should focus on examining the early life-stage of the fish rather than how long adults can live.

The CV of adult abundance always increased with increasing fishing mortality when a stochastic perturbation was introduced into the juvenile survival rate (Fig. 11, 12, 13). The results were somewhat surprising because I hypothesized that a stock with low resilience would have a higher autocorrelation in adult abundance, which would in turn increase the variance of the abundance. Contrary to this hypothesis, the CV of adult abundance always increased with increasing fishing mortality. Examination of the mean and standard deviation of the adult abundance showed that fishing mortality reduces the mean abundance, which reduces its standard deviation. If both were reduced at the same rate, the CV of adult abundance would remain the same, but the mean was reduced faster than the standard deviation. Consequently, the CV of adult abundance increased with increasing fishing mortality. Resilience is associated with the dominant eigenvalue of the linearized projection matrix, which measures a longer-term transient dynamics. Consequently, resilience appears to be a measure of intermediate time-scale dynamics, whereas the CV of adult abundance may be viewed as a measure of short time-scale dynamics. However, the CV is not necessarily a measure of how much a population fluctuates because it appears to be affected more by the equilibrium abundance.

An increase in the CV of adult abundance with increasing fishing mortality has been observed, and its potential cause was attributed to an increase in newly recruited individuals in the adult stage [21], which causes increased variance in the adult abundance. However, the results presented here show that an increase in the CV of adult abundance also occurs with semelparous fish, which comprise only newly recruited individuals in the adult stage. The cause of the increased CV of adult abundance is that the mean adult abundance is more sensitive than its standard deviation, and the sensitivity increases with increasing adult duration.

The analyses in this study were based on a model containing various assumptions (see *Model* section). A simple model was intentionally used to provide general insights into how demographic diversity affects the response of fish stocks to fishing mortality. The model did not include factors that many fishery biologists may consider important in understanding fish population dynamics. Amongst these are the effect of age and/or size on population parameters, autocorrelation in environmental fluctuations, depensatory processes under low population density (the Allee effect), other types of compensatory density-dependent processes, differential effects of fisheries on different size classes, changes in parameters caused by interactions among populations (e.g. predation and competition), and changes in population parameters resulting from rapid evolution as a response to fishing pressures. The results presented here should form the basis for further investigations on how these other factors might affect the optimal management of fish stocks.
